# Association of Sleep and Circadian Activity Rhythm with Emotional Face Processing among 12-month-old Infants

**DOI:** 10.1038/s41598-018-21448-0

**Published:** 2018-02-16

**Authors:** Wanqi Sun, Shirley Xin Li, Guanghai Wang, Shumei Dong, Yanrui Jiang, Karen Spruyt, Jiefan Ling, Qi Zhu, Tatia Mei-Chun Lee, Fan Jiang

**Affiliations:** 1 Department of Developmental and Behavioral Pediatrics, Shanghai Children’s Medical Center, Shanghai Jiaotong University School of Medicine, MOE-Shanghai Key Laboratory of Children’s Environmental Health, 1678 Dong Fang Road, Shanghai, 200127 China; 2 Department of Psychology, Sleep Research Clinic and Laboratory, The University of Hong Kong, Pokfulam Road, Hong Kong, Hong Kong SAR; 3 The State Key Laboratory of Brain and Cognitive Sciences, The University of Hong Kong, Hong Kong, Hong Kong SAR; 4 Lyon Neurosciences Research Center, INSERM U1028-CNRS 5292 - Waking Team, University Claude Bernard, School of Medicine, Lyon, France; 5 Department of Psychology, Laboratory of Neuropsychology, The University of Hong Kong, Pokfulam Road, Hong Kong, Hong Kong SAR

## Abstract

Sleep and circadian rhythmicity both play an important role in human’s cognitive functioning, yet the way in which early development of sleep and circadian rhythm affects cognitive processes and social learning in infants remains less understood. We examined the association of sleep and circadian activity rhythm (CAR) with face and emotional information processing in 12-month old infants. Face processing was measured by eye tracking, whereby infants’ scanning patterns and pupil dilations were calculated when they were presented with neutral, pleasant and unpleasant faces. Infants with better sleep quality (i.e., less waking after sleep onset) and lower sleep-wake pattern variability (i.e., higher inter-daily stability) exhibited a higher eyes over mouth fixation ratio (EMR). Infants with longer total sleep time showed larger pupil diameter changes in response to emotional facial expressions, more closely resembling the responses of adults. Our findings suggest the role of sleep and circadian rhythm in waking cognition and have implications for understanding the early development of social learning in young children.

## Introduction

Sleep is a complex, dynamic state regulated by the interaction between wake-dependent homeostatic sleep pressure and circadian timing system^1^, which is largely controlled by a central circadian pacemaker located in the suprachiasmatic nucleus (SCN) of the hypothalamus. Meanwhile, change in sleep may lead to altered circadian features^2^. Sleep and circadian rhythmicity both play an important role in human’s cognitive functioning, such as executive functions^3,4^. Executive functions may serve as the basis for social cognition and self-regulation, such as emotion regulation^5,6^. Accumulating evidence suggests the effects of sleep and circadian rhythm on emotion regulation and mental well-being^7,8^. Poor sleep quality and sleep deprivation have been found to impair facial emotion recognition in adolescents and adults^9,10^. Individuals with late chronotype, i.e., a circadian preference for later bedtime and rise time, have been found to show an increased sensitivity to sad facial expression^11^. These findings suggest that sleep and circadian rhythm may be implicated in cognitive and emotional processing, such as emotional face perception, the initial step of emotion regulation^12^.

Human newborns are most likely to hold an innate preference for face-like patterns and various communicable cues, which enable them to adapt to the social world efficiently in early life^13^. A few months after birth, infants are able to understand emotions^14^ and intentions of others^15^. Such a rapid development of social cognition seems difficult to achieve solely during the waking state, as infants spend a great proportion of time sleeping during this critical window of socio-emotional development. Several lines of evidence suggest the potential role of sleep and sleep-wake rhythmicity in early social brain development. For example, newborns and young infants show an ability to directly encode social information during sleep^16,17^. Sleep has also been shown to consolidate infants’ episodic memories from prior waking periods^18^, especially the memory from social interactions (e.g., representations of the mother and language rules)^19^. Evidence from basic science research has indicated the adverse impacts of circadian disruption on brain functions and behaviors^20^. Previous infant studies also suggested that better sleep-wake regulation, as reflected by a higher amplitude of sleep-wake cyclicity at birth, is predictive of a child’s later ability to control emotional reactivity and behaviors in social situations^21,22^. Specifically, infants including preterm babies with more robust sleep-wake cycle at 37 weeks of gestational age showed better emotional regulation in arm restraint paradigm at 6 months and separation-reunion paradigm at 12 months. Moreover, early sleep-wake regulation has been found to be associated with the mother-infant synchronization in face-to-face interaction^22,23^. Behavioral synchronization between infants and mothers might in turn facilitate the entrainment of circadian rhythm during its critical period of first 2–3 months of life^24,25^. Circadian rhythm regularity during infancy has also been reported to be predictive of the anxiety level in childhood^26^. It was found that one-month-old infants with more regular daily routine were less anxious even at 13 years of follow-up.

Infancy is an important developmental stage because socio-emotional abilities develop rapidly during this period of time, along with the acquisition of gross motor skills^27^ and the maturation of brain circuits^28,29^. Moreover, socio-emotional abilities during infancy may have long-term implications for later emotional well-being^30^. Despite some data suggesting a link between sleep and cognition among young children, direct evidence on how sleep facilitates infant’s development of social cognition, such as face processing, is limited. A previous study has found that infants with longer reported sleep duration show a stronger preference toward socially enriched stimuli (e.g., human faces) at 6 months old^31^. However, several questions remain untested, such as whether sleep affects infant’s sensitivity toward different emotional faces, the most important social stimuli, and how different aspects of sleep (e.g., sleep quality, sleep quantity) may affect face processing.

Here we examined whether sleep characteristics and circadian rhythm in infants as measured by actigraphy are associated with their emotional face processing, which could be indicated by the visual scanning patterns and pupillary reactivity when viewing neutral, pleasant and unpleasant facial expressions. As a baby-friendly measurement, pupillary reactivity can be used to assess physical arousal when processing emotional information^32^. Moreover, pupillary reactivity has been shown to be sensitive to the effects of sleep loss^33–35^. In addition to physical arousal, the scanning pattern of faces could reflect another important aspect of face processing. In particular, gazing into eye regions has often been emphasized in the child development literature, as the time spent looking into eyes is associated with the ability to recognize facial emotions^36^. As such, the eye tracking technique may serve as a useful tool to further investigate emotional face processing in relation to sleep and circadian rhythm in infants, and to expand our understanding on how early sleep and circadian development affect social cognition. We hypothesized that infants with longer sleep duration, better sleep quality and more regular sleep-wake circadian activity rhythm would show better social-cognitive responses, as reflected by showing more attention toward eye regions during face processing; while infants with shorter sleep duration, poorer sleep quality and more irregular circadian rhythm would exhibit altered pupillary reactivity when viewing emotional faces.

## Results

Fifty-two 12-month-old infants (age range: 11.73–13.17 months; 48.1% were boys) with complete actigraphic and eye-tracking data were included in this report. Sample characteristics, including sleep and circadian activity rhythm are shown in Table 1. The majority of the families participated in this study were from the middle-to-high socio-economic class (88.5% of mothers and 90.4% of fathers had an educational level higher or equal to a college degree). About 23% of the infants were receiving breastfeeding at the time of the study. Feeding pattern was not found to be associated with the key indices on sleep, circadian activity and face processing characteristics in the current sample. Most infants (94.2%) in the current sample shared bedroom with their parents or caregivers, and U test suggested there were no differences in the sleep parameters between co-sleepers and solitary sleepers. Maternal mood state was measured at 12 months by the Total Mood Disturbance (TMD) score from the Profile of Mood State (POMS). TMD score can be ranged from −32 to 200. The score of TMD in the current sample ranged from −14 to 101.

**Table 1:** Descriptive analysis on socio-demographic characteristics, sleep and circadian activity rhythm in infants.

	Mean or N	SD or %
***Socio-demographic characteristics***		
Age (months)	12.1	0.24
Male	25	48.1
Paternal educational level		
High school and below	5	9.6
College	40	76.9
Postgraduate	7	13.5
Maternal educational level		
High school and below	6	11.5
College	36	69.2
Postgraduate	10	19.2
Maternal mood state at 12-month^a^	27.74	29.79
Current breastfed	12	23.1
Bedroom sharing	49	94.2
***Sleep-related parameters***		
Total sleep time (min)^b^	665.66	47.68
Daytime sleep time (min)	119.59	42.28
***Characteristics of nighttime sleep***		
Nighttime sleep time (min)	546.06	44.13
Sleep onset latency (min)	10.79	11.44
Wake after sleep onset (min)	18.68	12.42
Nighttime sleep efficiency (%)	92.59	3.71
***Circadian-related parameters***		
Inter-daily stability	0.58	0.10
Intra-daily variability	0.87	0.14
Lowest 5 h activity	6245.61	3466.37
Lowest 5 h activity onset time	1.97	2.05
Most 10 h activity	211557.69	52861.05
Most 10 h activity onset time	9.76	1.51
Relative amplitude	0.94	0.03

^a^Maternal mood state was measured by the Total Mood Disturbance score from the Profile of Mood State.

^b^Total sleep time was calculated as the sum of daytime and nighttime sleep time.

### Infants’ Face Processing Characteristics at 12-month

Face processing characteristics in 52 healthy infants are presented in Table 2. Among infants, the total fixation duration on the face area did not differ across the three emotional expressions, including neutral, unpleasant and pleasant faces [*F*(2, 102) = 0.78, *P* = 0.460]. Two main parameters were used to analyze emotional face processing: (1) The eye/mouth ratio (EMR) was calculated as the eye-viewing proportion relative to the fixation duration on eyes and mouth areas, which reflected infants’ scanning patterns on faces. (2) The pupil size change was used to examine infants’ physical arousal when viewing the emotional faces. There were no gender differences in EMR and pupillary reactivity in infants.

**Table 2:** Descriptive analysis on face processing characteristics among infants (mean, SD).

	Neutral	Unpleasant	Pleasant	*F, P*
Fixation duration on whole face	2.42 (1.02)	2.45 (1.15)	2.34 (1.01)	0.78 (0.460)
Fixation duration on eye area (s)	0.61 (0.77)	0.59 (0.76)	0.65 (0.65)	**4.12** (**0.019)**^a^
Fixation duration on mouth area	0.19 (0.27)	0.28 (0.34)	0.32 (0.40)	1.51 (0.225)^a^
Eye/mouth ratio	0.63 (0.36)	0.57 (0.38)	0.60 (0.38)	1.66 (0.195)
Pupil size change 0–2 s (mm)	−0.25 (0.16)	−0.25 (0.14)	−0.32 (0.18)	**11.30** (**<0.001)**
Pupil size change 2–5 s (mm)	−0.07 (0.18)	−0.12 (0.15)	−0.13 (0.19)	**3.39** (**0.037)**
Pupil size change 0–5 s (mm)	−0.14 (0.15)	−0.17 (0.14)	−0.20 (0.17)	**4.83** (**0.010)**

^a^Statistical analysis was conducted on log-transformed data.

Significant results are marked in bold.

**Figure 1:**
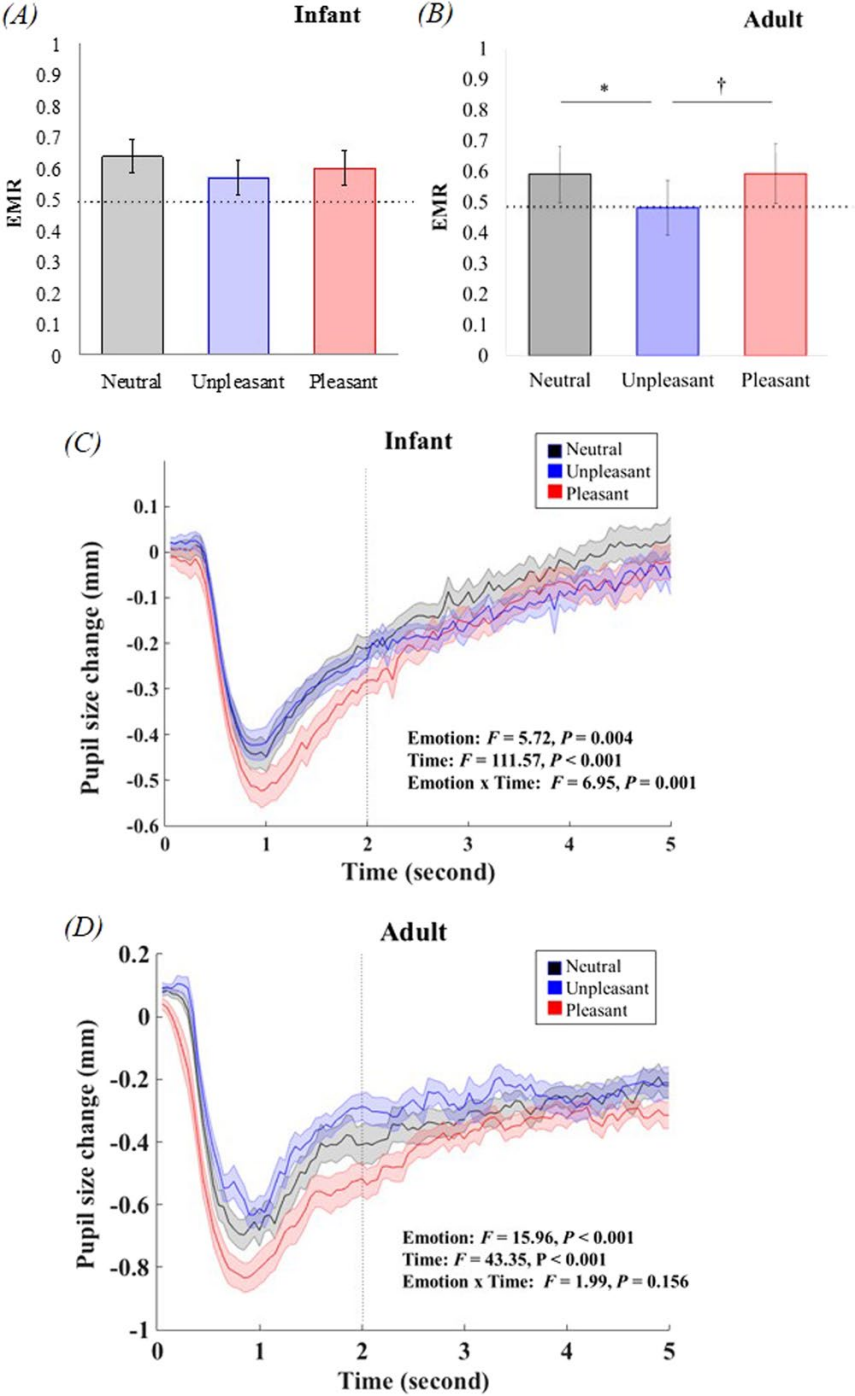
Face processing among infants and adults. (**A**) Infants showed no significant difference in EMR when scanning different emotional faces. (**B**) Adults showed lower EMR approaching borderline significance in unpleasant condition. (**C**) Average pupil size change in infants in three emotional conditions. (**D**) Average pupil size change in adults in three emotional conditions. Pupil size change in neutral condition is presented in grey, unpleasant condition is presented in blue, and pleasant condition is presented in red. EMR: eye/mouth ratio. Error bars represent standard error (SEM). *P = 0.040, ^†^*P* = 0.095.

As shown in Fig. 1A, the EMR of infants was not significantly different across three emotional conditions [*F*(2, 92) = 1.66, *P* = 0.195]. In neutral condition, infants spent more time on eyes than on mouth [*t*(50) = 2.98, *P* = 0.004]. This association was borderline significant for pleasant condition [*t*(49) = 1.89, *P* = 0.065], and non-significant for unpleasant condition [*t*(48) = 1.13, *P* = 0.263]. The face processing task was also tested in 15 healthy young adults to allow for a comparison with the well-developed ability of emotion discrimination^33^. Repeated measure ANOVA suggested a main effect of emotion on EMR approaching borderline significance in adults [*F*(2, 26) = 2.89, *P* = 0.074, LSD post-hoc: unpleasant < neutral, *P* = 0.040, Fig. 1B].

Infants’ pupil size change across three emotional conditions is shown in Fig. 1C. Since the initial light reflex and later phase of pupillary reactivity might involve different cognitive processes, such as alertness, active processing^37^ and emotional arousal^38^, we conducted a 3 (emotion) × 2 (time courses: 0–2 s vs. 2–5 s) repeated measure ANOVA to examine infants’ physical arousal when viewing the faces. The results indicated significant main effects of emotion [*F*(2, 102) = 5.72, *P* = 0.004; LSD post-hoc: neutral > pleasant] and time course [*F*(1, 51) = 111.57, *P* < 0.001] as well as the interaction between time course and emotion [*F*(2, 102) = 6.95, *P* = 0.001] on pupillary reactivity. Among adults, the main effects of emotion and time course on pupillary reactivity were significant [*F*_emotion_(2, 28) = 15.96, *P* < 0.001, LSD post-hoc: unpleasant > neutral > pleasant; *F*_time_(1, 14) = 43.35, *P* < 0.001], but the interaction between these two factors was not significant [*F*(2, 28) = 1.99, *P* = 0.156; Fig. 1D]. Moreover, a larger amplitude of the initial light reflex was not purely the result of luminance because: (1) the luminance measure of pleasant faces was not significantly higher than that of the other two emotional conditions (Supplementary Information), and (2) the greater initial change in pupil size disappeared when the faces were shown upside down (Fig. S2).

### Associations of Sleep and Circadian Activity Rhythm with Face Processing

The associations of sleep-related and circadian activity rhythm (CAR) parameters with face processing indicators in infants are shown in Table 3. The degree of maternal mood disturbance as measured by the Profile of Mood State was not associated with either face scanning pattern or pupil dilation of infants.

**Table 3:** Results of repeated measures GLMs on face processing characteristics (*F, P*).

	Sleep Variables			Circadian Variables			
	TST	WASO		IS	IV	RA	L5o
**DV: EMR**			**DV: EMR**				
Emotion	**3.56** (**0.033)**	1.33 (0.269)	Emotion	0.27 (0.766)	0.21 (0.808)	0.58 (0.561)	1.17 (0.315)
Emotion × Sleep	**3.58** (**0.032)**	0.30 (0.739)	Emotion × Circadian	0.46 (0.636)	0.28 (0.760)	0.64 (0.528)	0.15 (0.865)
Sleep	2.12 (0.120)	**5.79** (**0.020)**	Circadian	**10.56** (**0.001)**	1.77 (0.191)	**7.16** (**0.010)**	**8.00** (**0.007)**
**DV: Pupil Size Change**			**DV: Pupil Size Change**				
Emotion	0.31 (0.878)	2.13 (0.124)	Emotion	0.27 (0.762)	0.46 (0.630)	0.17 (0.848)	2.70 (0.072)
Emotion × Sleep	0.14 (0.871)	0.04 (0.962)	Emotion × Circadian	0.85 (0.432)	0.11 (0.893)	0.18 (0.834)	0.95 (0.389)
Time	0.15 (0.700)	**52.21** (**<0.001)**	Time	0.001 (0.974)	**4.38** (**0.041)**	1.13 (0.294)	**48.93** (**<0.001)**
Time × Sleep	0.13 (0.725)	2.53 (0.118)	Time × Circadian	3.87 (0.055)	0.16 (0.693)	2.04 (0.160)	0.64 (0.427)
Emotion × Time	0.80 (0.451)	1.73 (0.182)	Emotion × Time	2.06 (0.132)	1.25 (0.292)	0.07 (0.938)	**4.33** (**0.016)**
Emotion × Time × Sleep	0.56 (0.575)	0.20 (0.816)	Emotion × Time × Circadian	2.28 (0.107)	0.76 (0.471)	0.09 (0.910)	0.48 (0.621)
Sleep	**7.59** (**0.008)**	0.01 (0.942)	Circadian	0.05 (0.827)	0.02 (0.886)	0.03 (0.856)	0.05 (0.817)

Each pair of dependent variable (DV) and sleep/circadian parameter was entered into a single general linear model (GLM). EMR: eye/mouth ratio; TST: total sleep time; WASO: wake after sleep onset; IS: inter-daily stability; IV: intra-daily variability; RA: relative amplitude; L5o: lowest 5 h activity onset time. Significant results are marked in bold.

**Figure 2:**
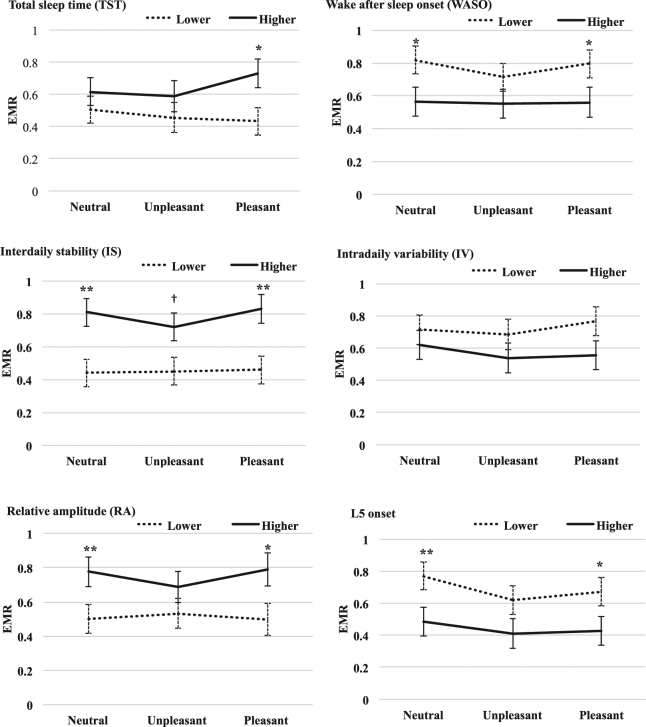
Face scanning pattern among infants in the higher and lower tertile of sleep and circadian characteristics. EMR: eye/mouth ratio. Error bars represent standard error. ^†^*P* < 0.1, *P < 0.05, **P < 0.01.

Repeated measure general linear models (GLM) suggested that EMR was correlated with several sleep and circadian-related parameters (Table 3). There was a significant interaction between TST and emotional condition [*F*(2,90) = 3.58, *P* = 0.032], but the main effect of TST was not significant [*F*(1, 45) = 2.12, *P* *=* 0.120]. There were significant main effects of WASO [*F*(2, 90) = 5.79, *P* = 0.020], IS [*F*(2, 90) = 10.56, *P* = 0.001], RA [*F*(2, 90) = 7.16, *P* = 0.010] and L5o [*F*(2, 90) = 8.00, *P* = 0.007] on EMR, while the interactions between these sleep/circadian parameters and emotion were not significant. Neither the main effect of IV nor its interaction with emotion on EMR was significant. Infant participants were further categorized for analyses according to the tertiles of the major sleep and circadian-related parameters (Fig. 2). Subgroup analyses suggested that infants with longer TST had higher EMR in pleasant condition [*t*(33) = −2.58, *P* = 0.015]. Infants with less WASO had higher EMR in neutral condition [*t*(31) = 2.30, *P* = 0.031] as well as pleasant condition [*t*(31) = 2.19, *P* = 0.040]. Infants with higher IS had higher EMR in neutral condition [*t*(32) = −3.06, *P* = 0.005] as well as pleasant condition [*t*(31) = −3.01, *P* = 0.005]. There was a borderline significant difference for IS in the unpleasant condition [*t*(31) = −2.01, *P* = 0.054]. Infants with higher RA showed higher EMR in both neutral [*t*(33) = −3.12, *P* = 0.004] and pleasant conditions [*t*(32) = −2.71, *P* = 0.011]. Infants with earlier L5o showed higher EMR in both neutral [*t*(33) = 2.80, *P* = 0.009] and pleasant conditions [*t*(32) = 2.32, *P* = 0.028].

**Figure 3:**
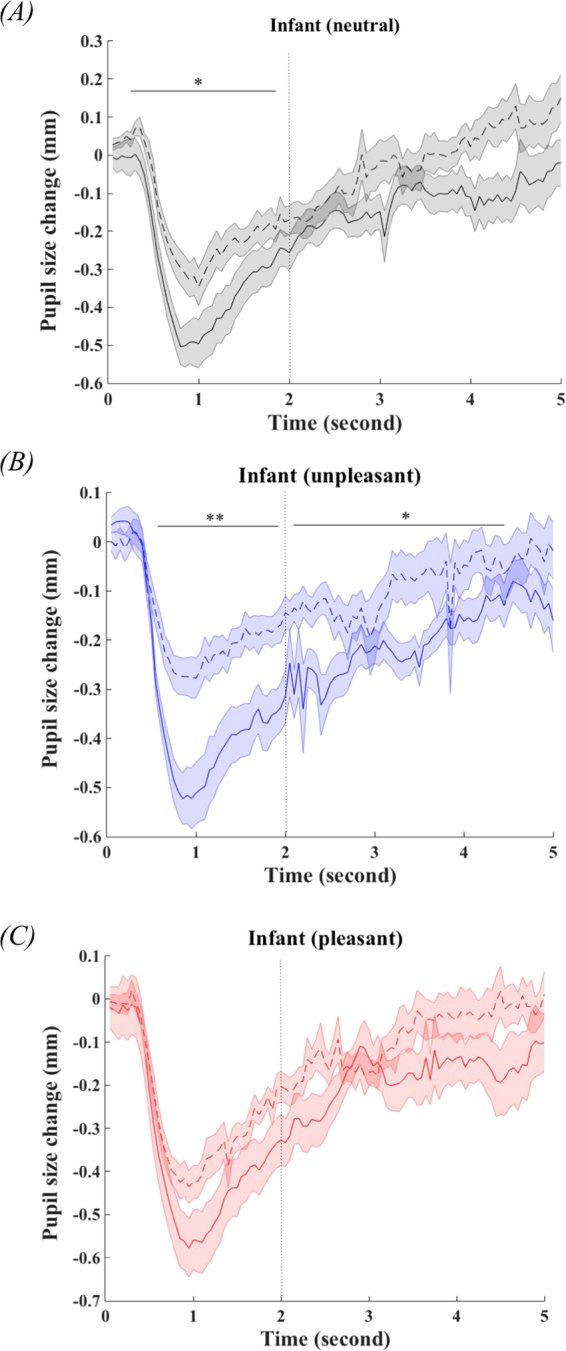
The comparisons of pupillary reactivity among infants in the higher (solid line) and lower (dash line) tertile of total sleep time (TST). Error bar represent standard error. *P < 0.05, **P < 0.01.

Pupillary reactivity was only correlated with total sleep time [*F*(1, 50) = 7.59, *P* = 0.008], while none of the interactions was significant in the repeated measure GLM (Table 3). The comparisons of pupillary reactivity between infants in the higher and lower tertile of total sleep time are shown in Fig. 3. Subgroup analyses suggested that infants with longer TST had larger pupil size change in the initial phase of neutral condition [*t*(34) = 2.41, *P* = 0.022], and in the initial and later phase of unpleasant condition [*t*_0–2s_(34) = 3.42, *P* = 0.002; *t*_2–5s_(34) = 2.30, *P* = 0.028]. There was no significant difference in pupillary reactivity in the pleasant condition between infants with longer and shorter TST.

## Discussion

To our knowledge, this is the first study to show that sleep and circadian rhythm are implicated in infants’ face processing. We found that infants’ face scanning pattern, as reflected by their preference toward eye region, was related to several sleep- and circadian-related parameters, such as sleep quantity, sleep quality, circadian stability, circadian amplitude and circadian phase. Infants with better sleep quality and more stable circadian activity rhythm showed a higher preference toward the eye region of the faces, while infants with shorter sleep duration showed a lower preference toward the eye region. Regarding physical arousal when viewing emotional faces, pupillary reactivity was mainly related to habitual sleep quantity. That is, infants with shorter sleep duration exhibited less physical arousal, especially when viewing unpleasant faces.

In line with previous research, the current study found that 12-month-old infants showed a preference toward eyes when viewing static neutral faces. A differential eye preference toward different emotional expressions was also found to emerge at this age. Making eye contact is an important basis in real life social interaction, as eyes usually convey information of other’s mental state (e.g., emotions) or signals for social reference (e.g., dangers). Looking into the eyes could help infants to understand others and social context. Avoiding eye contact is one of the typical presenting symptoms related to deficits in social interaction in children with autism spectrum disorders (ASD)^39^. Although early scanning patterns may not necessarily be predictive of later socio-emotional development^40^, normal infants are expected to develop a stable preference toward eyes over mouth when viewing static faces as a result of early social experiences^41^. A previous study examining infants’ scanning patterns found that 7-month-old infants did not avoid direct eye contact when being shown either angry or fearful faces, whereas adults did spend shorter dwell time on eyes when viewing those threat-related facial expressions^42^. The current findings may extend our understanding of the time window of the early development of emotional facial perception in young children. A trend of avoiding eye contact with unpleasant faces was found in 12-month-old infants in this study.

Our findings on the associations of sleep and circadian rhythms with infants’ face scanning pattern complemented previous research, which showed that early sleep and circadian rhythm development could predict later attention allocation and orientation, especially in social contexts^43–45^. From a pathological perspective, sleep disturbances are commonly seen among children with socio-emotional developmental deficits, such as ASD^46^. Circadian rhythm dysregulation has been speculated to be the mediator between sleep and ASD, albeit limited data^47,48^. The current finding may provide some evidence to support the link between circadian sleep-wake patterns and the development of social cognition, although the exact underlying mechanisms remain untested. The observed associations of sleep quality and circadian rhythm with face scanning patterns could potentially reflect how sleep may affect social learning and memory of face representations. In this regard, previous research has consistently demonstrated the effect of sleep on memory consolidation^18^. Post-learning sleep has shown to consolidate memory related to social context in infants^19^ and improve face recognitions regardless of the emotional valence of the faces^49^. Therefore, it is possible that insufficient sleep, impaired sleep quality and poor circadian rhythmicity might lead to poor learning and memory of social cues (e.g. faces) in young children. Nonetheless, future longitudinal studies are warranted to further test this hypothesis.

In the present study, infants with different sleep duration showed differential pupillary reactivity when processing facial expressions. It is known that pupillary reactivity and baseline pupil size fluctuate depending on the level of alertness or vigilance^50^, which is often affected by sleep duration. Apart from the changes in automated ocular motor responses^51^, acute sleep deprivation could also lead to reduced or even altered affective-related pupil dilations^34^. In a previous study, the effect of sleep deprivation on emotional information processing was found to vary across different emotional valences; it particularly affects the reactivity in response to negative stimuli^33^. Although the interaction between sleep duration and emotion was not found to be significant in the GLM analysis of this study, there was a trend suggesting that emotional reactivity toward negative stimuli might be more affected by sleep duration in infants. In addition, a previous study conducted in adults showed that the use of emotional regulation strategies (e.g., distraction) during image presentation induced greater changes in pupil diameter after the initial light reflex, as compared with passively viewing the images^38^. It is possible that infants with longer sleep duration may have better emotion regulation abilities (e.g. self-soothing), and thus showed greater changes in pupil diameters during the negative image presentation.

Greater physical arousal was observed in infants when being shown pleasant faces. In the current study, the increased initial pupil size change during the presentation of pleasant faces was observed in both infant and adult groups. This effect was eliminated when the faces were presented upside down. Thus, the initial light reflex might be also explained by the modulation of emotional information^52^. Similarly, a previous study has observed that infants as early as 7 months old showed automatic unconscious discrimination of facial emotions^53^. However, when comparing with neutral emotions, infants showed larger pupillary reactivity to negative faces, while adults showed smaller responses. In addition, during the later phase of emotional information processing, infants were not able to maintain the discrimination between pleasant and unpleasant facial expressions. A previous study found that 6- and 12-month-old infants exhibit larger pupil diameters (smaller changes) after the initial light reflex when seeing other infants displaying negative emotions, as compared to watching those exhibiting positive emotions^32^. One possible explanation for the minor discrepancy in pupil diameters observed across different emotional expressions in the current study may be related to the differences in the presenting stimuli. For instance, video records involving both facial expressions and vocalizations (e.g., laughter and crying) were used in the previous study. The emotions presented in this way could be more intense and well-expressed than those conveyed by the static faces. Another possible explanation is that the brain circuits involved in emotional face processing, such as frontal and limbic systems, remain under-developed at 12-month^54^. Neuroimaging studies have shown that a distinct sensitivity to emotional information is likely caused by the differential diminished cortical-amygdala functional connectivity when processing positive and negative emotions^55^. Thus, the premature emotion discrimination abilities might be the result of the under-development of these essential neural circuits in infants^29^. In this regard, as the face stimuli could successfully trigger larger pupil diameter changes among adult subjects in this study, this observation suggested that infants’ ability to differentiate emotional facial expressions might be still rudimentary.

Some limitations of the current study should be noted. First, adults’ eye tracking data were used as a reference and greater pupil size change was considered suggestive of increased maturity. As evident in the present study, when compared with adults, infants’ pupillary reactions were generally lower across all emotional conditions. Nevertheless, larger pupil diameter in response to emotional faces measured at 9 months has been found to be predictive of poorer socio-communicative abilities at 18 months in a recent infant study^56^. The interpretation of eye tracking data in that study may be limited by using raw pupil size without baseline correction. Future studies applying more sophisticated neuroscience measures (e.g., functional near-infrared spectroscopy) and longitudinal design are warranted to make a more accurate interpretation of face processing characteristics during infancy. Secondly, the stimuli used in the current study were not natural human faces, and the facial expressions only involved a limited range of emotions and were not the typical ones (e.g., angry, fearful, sad) commonly used in the studies conducted in infants. Although young children are generally not expected to be able to differentiate a wide range of specific negative expressions, such as anger, fear and disgust^57^, the use of un-natural human faces might potentially increase difficulties for infants to discriminate emotions. Nonetheless, this effect was controlled by presenting the same set of faces to all the infants in current study. Thirdly, the current study was limited by a cross-sectional design, which precluded inferring causal relationships. While previous studies using nap deprivation paradigm have shown that acute sleep lose could alter emotional reactivity among toddlers^58,59^, the mechanistic relationship between sleep and face processing among younger children remained untested. Further studies are warranted to explore the potential mechanism underlying the association between sleep, circadian rhythm and socio-emotion information processing among infants.

## Conclusions

We found an association of sleep and circadian rhythm characteristics with waking social cognitions in infants, particularly face processing, an important predictor for socio-emotional functions. These findings may provide support for the importance of adequate and regular sleep in facilitating early social learning in young children. Further research is needed to explore the potential mechanism underlying the relationship between sleep-wake pattern and socio-emotional development during infancy.

## Methods

### Participants

Participants were healthy infants from a prospective birth cohort recruited at Shanghai Children’s Medical Center. Inclusion criteria at recruitment included: (1) full-term infants, without any complications at birth (defined as no history of admission to intensive care unit at birth), (2) free of maternal mental disorders during the prenatal period. Fifty-three infants from the birth cohort were randomly selected and invited to complete the face processing task. One infant was unable to complete the task due to fussiness during the experiment. The study protocol was approved by the Shanghai Children’s Medical Center Human Ethics Committee, and the study was performed in accordance with relevant guidelines and regulations. Written informed consent was obtained from the caregiver of each child. Participants received RMB 60 (US$1 = RMB7) as their travel allowance for taking part in this study.

### Stimuli and Procedure

A total of 12 face stimuli were generated using FaceGen software. There were four identities, all female, and each identity exhibited three emotions (i.e., neutral, pleasant and unpleasant). The unpleasant facial expression was a mixture of anger, fear and disgust (predominant) categorized by the software. The examples of faces used in the current study and the definition of eyes and mouth areas are shown in Fig. S1. Adult participants (n = 15) were recruited and completed the same experimental procedure as the infants. They were also asked to rate the valence of emotions. There were no significant differences in the luminance of stimuli across three expressions (see Supplementary Experimental Procedure).

Images (400 × 400 pixel) were presented in the middle of the screen (1366 × 768 resolution) through the Tobii TX300 eye tracker. Infants were seated on their parent’s lap approximately 60 cm from the screen, and their eye movements and pupil diameters were recorded at 60 Hz. Before entering the real experiment, a five-point calibration was achieved successfully within three attempts for each infant. Each image was presented once for five seconds in a random order. The same animation with sound (i.e., a smiling sun) was presented for three seconds between images.

Pupillometry was recorded throughout the experiment and averaged into consecutive 50-ms bins (i.e., 3 data points). Baseline correction was performed for each trial. The mean pupil diameter from the last accumulated second of the inter-trial gap was counted as the baseline. Pupil size change was calculated for each trial and averaged across three emotional expressions for each participant. Data on pupil size change was counted as missing if the infant did not attend to the screen during the presentation or inter-stimuli interval (because baseline pupil size could not be calculated). Taken together, pupil size change was not calculated in 32 trials (5%). Data reduction was completed by MATLAB.

In addition, as pupil size change could vary, potentially affected by the time of the day, all testing sessions were arranged between 8:30 am and 4:00 pm. Repeated measure ANOVAs suggested that there were no significant differences in pupillary reactivity and EMR between infants tested before and after 12:00 pm.

### Measures of Sleep and CAR

To assess infants’ habitual sleep pattern and circadian rhythm, infants wore an actigraphy (Actiwatch 2, Philips Respironics, USA) on their non-dominant ankle for 7 consecutive days (24-hour x 7 days) following the completion of the face processing task. The primary caregiver was asked to keep a sleep diary for the infant during the period while the infant was wearing actigraphy. To ensure the reliability of data, all infants had at least three days of valid objective sleep records^60^. All recording days were included in the analysis. The average recording period was 6.56 (*SD* 2.03) days, ranging from 3 days to 13 days.

Objectively measured sleep parameters (e.g., total sleep time, sleep efficiency and wake after sleep onset) were calculated using the Actiware (Version 6.0, Philips Respironics, USA). These sleep parameters were defined in accordance with previous practice recommendations^61^. The epoch length was set as 30 seconds, and the wake threshold was set as auto. Bedtime and rise time were entered into the software according to the data recorded in sleep diary. Sleep onset and offset criteria were set at 3 and 5 consecutive minutes, respectively. Total sleep time was calculated as the sum of daytime and nighttime sleep time. Calculation of sleep onset latency (minutes) and wake after sleep onset (minutes) was based on nighttime sleep. Sleep onset latency was calculated as the interval from bedtime (recorded in sleep diary) and sleep onset time (estimated by actigraphy).

Nonparametric circadian rhythm analysis (NPCRA) were applied to calculate CAR^62^. In the NRCRA, the average activity level in the least active contiguous 5 h (L5) and the most active contiguous 10 h (M10) and their onset time (i.e., L5o and M10o) were derived. An index describing magnitude was further calculated as relative amplitude [RA = (M10 − L5)/(M10 + L5)]. A larger number indicated a larger magnitude. Inter-daily stability (IS) and intra-daily variability (IV) were calculated to examine the regularity of infants’ circadian sleep-wake patterns. Higher IS indicated higher day-to-day invariability (ranging from 0–1), and higher IV indicated higher 24-h rest-active fragmentation (ranging from 0–2)^63^.

### Statistical analysis

An initial examination of the data was performed to test for normality, and the variables that were not normally distributed (i.e., fixation duration on eyes and mouth areas) were converted by log-transformation for further parametric test. The effects of emotion and time course on face processing characteristics including EMR and pupil size changes were examined by repeated measure ANOVAs.

Among the dozen of sleep and circadian parameters, 6 key indicators were chosen for further analysis. Total sleep time (TST) was used to indicate sleep quantity, wake after sleep onset (WASO) was used to indicate sleep quality, inter-daily stability (IS) and intra-daily variability (IV) were selected to reflect the stability and fragmentation of circadian rhythm, relative amplitude (RA) was selected to represent the circadian amplitude, and least active contiguous 5 h onset time was used to indicate the circadian phase. The effects of sleep and circadian rhythm on face processing were tested by repeated measure general linear model (GLM), in which continuous sleep parameters were entered as between subject factor.

Subgroup analysis was conducted when there was a significant main effect or interaction of sleep and circadian parameters on face processing to show the trend of correlation. Infants were further categorized according to the tertiles of each sleep and circadian parameters. Face processing patterns (i.e., EMR, pupil size change) were compared between infants with higher and lower third of sleep and circadian rhythm characteristics by student t-test, in separation of emotion and time course.

All analyses were performed using the Statistical Package for Social Sciences (SPSS) for Windows 22.0 (SPSS Inc., Chicago, IL, USA). The statistical significance level was set at 0.05.

### Data availability

The datasets generated during and/or analysed during the current study are available from the corresponding author on reasonable request.

## Electronic supplementary material

Supplementary information
