# Supplementary material for: Association of Sleep and Circadian Activity Rhythm with Emotional Face Processing among 12-month-old Infants
Source: Sci Rep. 2018 Feb 16;8:3200. doi: 10.1038/s41598-018-21448-0 (PMC5816664; doi:10.1038/s41598-018-21448-0)

Association of Sleep and Circadian Activity Rhythm with Emotional Face Processing among 12-month-old Infants

Wanqi Sun [1,2], Shirley Xin Li [2,3*], Guanghai Wang [1], Shumei Dong [1], Yanrui Jiang [1], Karen Spruyt [1,4], Jiefan Ling [2], Qi Zhu [1], Tatia Mei-Chun Lee [2,3,5*], Fan Jiang [1*]

[1] Department of Developmental and Behavioral Pediatrics, Shanghai Children's Medical Center, Shanghai Jiaotong University School of Medicine, MOE-Shanghai Key Laboratory of Children’s Environmental Health, 1678 Dong Fang Road, Shanghai, 200127, China.
[2] Sleep Research Clinic and Laboratory, Department of Psychology, The University of Hong Kong, Pokfulam Road, Hong Kong SAR.
[3] The State Key Laboratory of Brain and Cognitive Sciences, The University of Hong Kong, Pokfulam Road, Hong Kong SAR.
[4] Center of Research in Neurosciences of Lyon, Faculty of Medicine, The Université Claude Bernard Lyon 1, INSERM.
[5] Laboratory of Neuropsychology, The University of Hong Kong, Pokfulam Road, Hong Kong SAR

* To whom correspondence should be addressed.

Fan Jiang, MD, PhD. Department of Developmental and Behavioral Pediatrics, Shanghai Children's Medical Center, 1678 Dong Fang Road, Shanghai, China, 200127, fanjiang@shsmu.edu.cn, +86 (021) 58750573.

Shirley Xin Li, PhD, DClinPsy. Department of Psychology, The University of Hong Kong, Pokfulam Road, Hong Kong. shirleyx@hku.hk, +852 39177035.

Tatia Mei-Chun Lee, PhD. Department of Psychology, The University of Hong Kong, Pokfulam Road, Hong Kong. tmclee@hku.hk, +852 39178394.

Supplementary Information

Supplementary information on testing materials

The intensity of emotions was controlled by the *FaceGen* software (Singular Inversions, Inc. CA) and was verified by an adult control group. Fifteen university students participated in the same experiment arranged in the afternoon and were also asked to rate the arousal and valence level for each image on the visual analogue scales (VAS). Adult ratings suggested that the facial expressions were well differentiated in terms of valence [$F(2,177) = 247.01$, $P < 0.001$] and arousal [$F(2,174) = 49.96$, $P < 0.001$]. Similar to the findings among infants, neither the total fixation duration on faces [$F(2, 28) = 2.65$, $P = 0.089$] nor the fixation ration on eye/mouth [$F(2, 26) = 2.89$, $P = 0.074$] was different across emotions. The 3 (emotions) x 2 (time courses) repeated measure ANOVA indicated significant main effects of emotion on pupil size change [$F(2, 28) = 15.96$, $P < 0.001$; LSD post-hoc: unpleasant > neutral > pleasant] and time course [$F(1,14) = 43.35$, $P < 0.001$], but the interaction between time course and emotions was not significant [$F(2, 28) = 1.99$, $P = 0.156$]. Moreover, average pupil size change (i.e., average of 5 s) was negatively associated with valence (Pearson's $r = -0.29$, $P < 0.001$) but not arousal (Pearson's $r = -0.03$, $P = 0.727$).

Meanwhile, the luminance of images was also measured and well-controlled. The results of Kruskall-Wallis test indicated that there was no significant difference in luminance across three emotional expressions (Chi-square = 1.92, $P = 0.383$; neutral: M = 64.9 cd/m^2, rank = 4.88; pleasant: M = 79.6 cd/m^2, rank = 8.38; unpleasant: M = 71.2 cd/m^2, rank = 6.25).

To further rule out the potential confounding effect of luminance on pupil size change, adult participants were presented with the reversed faces after completing the same experiment as infants. As shown in Fig. S2 the initial enhanced pupillary reaction to pleasant faces disappeared after the same set of faces was reversed, while the pupil size change during the later phase of presentation remained consistent.

Fig. S1. Examples of faces used in the current study and the definition of eyes and mouth areas.

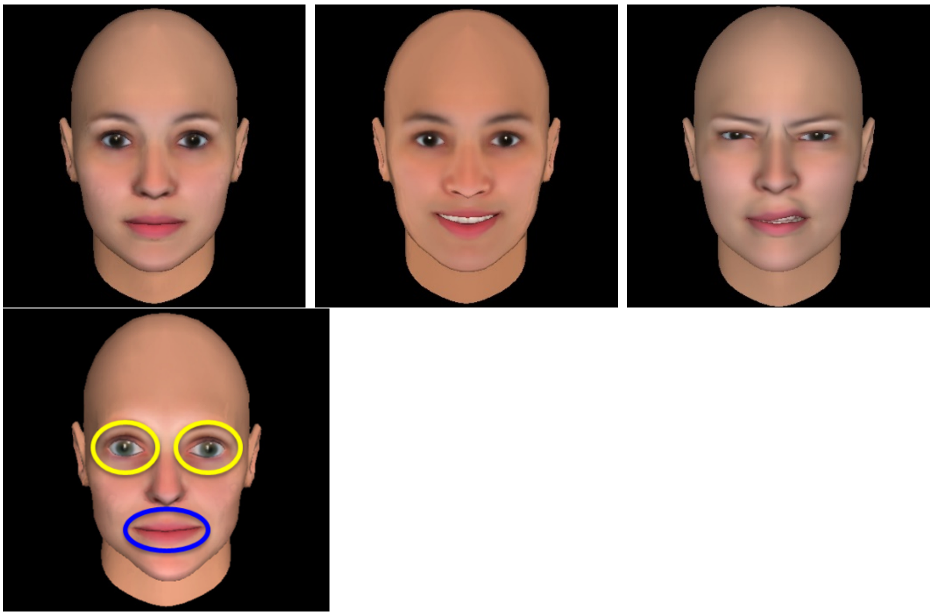

Fig. S2. Average pupil size change in adults in three emotional conditions when faces were upside down.

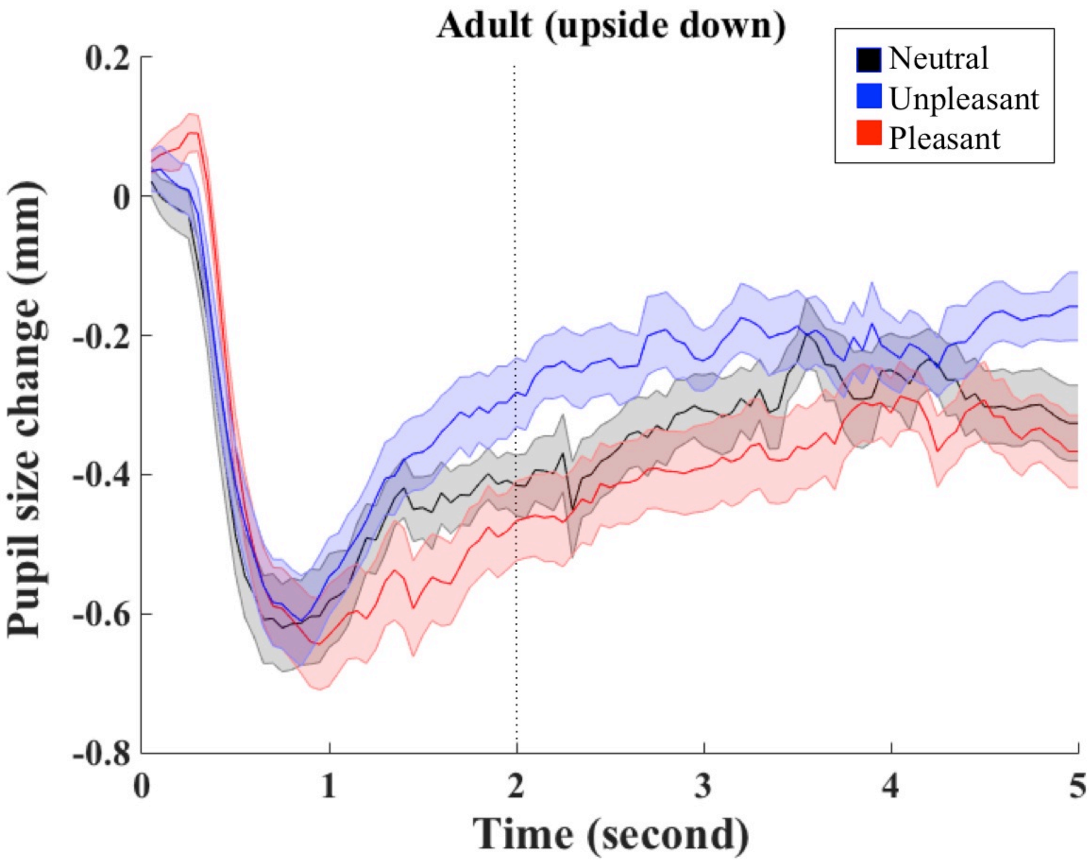

Supplement: Supplementary file 1 — Supplementary information [file 41598_2018_21448_MOESM1_ESM.pdf]
